# Impact on women's body satisfaction of exposure to postpartum imagery on social media

**DOI:** 10.3389/fdgth.2025.1379337

**Published:** 2025-03-12

**Authors:** Megan L. Gow, Maddison Henderson, Amanda Henry, Lynne Roberts, Heike Roth

**Affiliations:** ^1^ The George Institute for Global Health, University of NSW, Sydney, NSW, Australia; ^2^ School of Population Health, UNSW Medicine and Health, Sydney, NSW, Australia; ^3^ The University of Sydney Children’s Hospital Westmead Clinical School, The University of Sydney, Sydney, NSW, Australia; ^4^ Women's and Children's Health, St George Hospital, Sydney, NSW, Australia; ^5^ Discipline of Women’s Health, School of Clinical Medicine, UNSW Medicine and Health, Sydney, NSW, Australia; ^6^ St George and Sutherland Clinical Campus, School of Clinical Medicine, UNSW Medicine and Health, Sydney, NSW, Australia; ^7^ Faculty of Health, University of Technology Sydney, Sydney, NSW, Australia

**Keywords:** postpartum, social media, Instagram, health promotion, body image, body satisfaction, thin-ideal

## Abstract

**Background:**

Social networking sites may be a convenient, accessible and low-cost option for delivering health information at scale to postpartum women. However, social media use is associated with decreased body satisfaction and may contribute to psychological ill-health. Our study aimed to determine whether exposure to body-focused imagery, typical of imagery targeting postpartum women on Instagram, is associated with a reduction in state body satisfaction and state body appreciation. Secondly, we aimed to determine whether including postpartum-health-focused imagery, in conjunction with body-focused imagery, is associated with improving state body satisfaction/appreciation, compared with no postpartum health content.

**Methods:**

A single blinded quasi-experimental survey study, recruiting women who had given birth in the previous 2-years, asked participants about key demographic information, social media use and assessed thin-ideal internalization and media appearance pressures using validated tools. Participants were then exposed to either (1) 15 body-focused images of women with a thin-average level of adiposity; (2) as per (1) PLUS 5 postpartum-health-focused images; or (3) as per (1) PLUS 15 postpartum-health-focused images. State body satisfaction/appreciation were assessed before and after image exposure.

**Results:**

State body satisfaction/appreciation did not change from pre- to post-image exposure in any groups and measures were not different between groups at any time point.

**Discussion:**

Short-term exposure to body-focused imagery typical of Instagram content targeting postpartum women may not alter state body satisfaction or state body appreciation. Furthermore, incorporating postpartum-health-focused imagery did not alter results. Further research investigating whether an intervention providing health information to postpartum women via social media platforms improves health outcomes may be warranted.

## 1 Introduction

Given their convenience, accessibility, and low cost, women of reproductive age are utilizing social media platforms to access health information that was previously provided by healthcare professionals, printed media or friends and family (1). One major platform is the photo and video sharing application, Instagram. It is estimated that 39% of Instagram's billion monthly active users are females of childbearing age, aged 18–44 years (2). Our recent survey study found that 92% of Australian postpartum women (with a child <2 years of age) had an Instagram account and 81% reported accessing Instagram more than once per day (3). Therefore, Instagram presents a potentially useful avenue for conveying relevant health information to women postnatally, such as information about nutrition, exercise, psychological wellbeing and breastfeeding.

However, evidence exists suggesting associations between social networking site use and increased body dissatisfaction and decreased mood in young women (4–7). Body dissatisfaction refers to negative subjective evaluations of one's own body size and shape, arising when there is a discrepancy between one's own body image and ideals (8). Reduced body satisfaction among Instagram users has been attributed to the carefully curated images uploaded to Instagram by users that may present an “ideal” rather than “real” version of themselves (9).

This potential impact of social networking site use on body image and body dissatisfaction may be particularly relevant for women in the postpartum period who, in comparison to women during pregnancy, are more likely to be experiencing body dissatisfaction that worsens over time (10). This increase in body dissatisfaction is likely due to women viewing their bodies less as a nurturing entity over time since childbirth, and an increase in the tendency to appraise their bodies in comparison to their pre-pregnancy size and shape (11). Hence, it is possible that exposure to imagery on platforms such as Instagram may exacerbate this vulnerability for psychological ill-health, leading to worsening body dissatisfaction and further problems including postnatal depression (12). Such negative impacts on the mental health of mothers may reduce engagement in practices, such as breastfeeding, exercise and healthy eating, that can assist in optimizing the physical and mental health of both the mother and the child (13).

If social networking sites are to be used for delivering health information to postpartum women, it is important to understand how encouraging women to engage more with social networking sites to access health information may impact their body satisfaction. Therefore, this study aimed to determine how exposure to typical body-focused imagery affects state body satisfaction and state body appreciation in postpartum women. Secondarily, we aimed to determine how the inclusion of health-information-focused imagery on social networking sites, in conjunction with body-focused imagery, affects state body satisfaction and state body appreciation, compared with body-focused imagery only. We hypothesized that exposure to body-focused imagery alone would reduce body satisfaction and appreciation, but that inclusion of health-information-focused imagery in conjunction with body-focused imagery would attenuate these reductions. This hypothesis is based on Objectification Theory which refers to the internalization of body image concerns of girls and women that depend largely on how they think others see and judge them (14).

## 2 Methods

Single (participant) blinded quasi-experimental study. Inclusion criteria were: Australian-residing adult (≥ 18 years) women who had given birth within the last 2 years and had access to a phone or computer with internet, including postpartum women who were once again pregnant. Women with low English proficiency were excluded due to the survey being in English and interpreter services not available for this study. This study was approved by The University of Sydney's Human Research Ethics Committee on 24 June 2022 (protocol number: 2022/148).

Recruitment occurred from 3 August to 7 November 2022 via advertisements on social networking sites (Facebook, Instagram, Twitter/X), Playgroup New South Wales (a not-for-profit Australian organization that helps connect families and children to people and services in the community to positively impact their quality of life) newsletters and paid text messaging advertisements to Playgroup New South Wales members. Snowball sampling was used, whereby participants who completed the questionnaire were asked to share study information with eligible friends/family/associates.

To prevent potential response bias, participants were not informed of the quasi-experimental nature of the study but were simply informed that they would answer questions including a set about body image before and after viewing a series of images taken from Instagram (i.e., participants were blinded to the experimental conditions). Survey responses were non-identifiable.

### 2.1 Interventions

Consenting participants were exposed to one of three sets of images (Table 1) with each image being displayed for 7 s. Study investigators regularly monitored the survey completion rates to ensure an equal number of participants were recruited to Group 1, Group 2 and Group 3. The survey had to be manually switched from Group 1, 2 or 3 for practical reasons related to limitations of REDCap to perform randomization. The images used for this study included 15 Instagram images, originally sourced from the hashtag #postpartumbody, that were identified and described in detail in a content analysis where images were rigorously coded for adiposity, muscularity, pose and attire (15). It is unknown whether images used were “idealized” in any way (e.g., whether filters were used for photographs). Chosen images had 100% agreement between coders, and were representative of images typically associated with this hashtag. Notably, images were representative of societal ideals (i.e., thin-average adiposity, little-to-none or visible muscularity, non-specific, sexy or fitness-focused pose and nearly half in active wear/fitness attire) and were not necessarily representative of the spectrum of typical postpartum body types (i.e., only 5% of images were featuring a postpartum body feature such as stretch marks, and only 9% were images of women with high adiposity) (15).

**Table 1:** Image exposure of three intervention groups.

Study group	Body-focused imagery	Postpartum-health-focused imagery
Group 1	15 images of women with a thin-average level of adiposity^a^	Nil
Group 2	15 images of women with a thin-average level of adiposity^a^	5 images focused on various aspects of postpartum health
Group 3	15 images of women with a thin-average level of adiposity^a^	15 images focused on various aspects of postpartum health

^a^ Images sourced from those characterized in content analysis of images tagged with #postpartumbody (13).

Health information images were sourced from relevant hashtags, e.g., #postpartumhealth, #infantfeeding, #postpartumdepression, #postpartumdiet, #postpartumselfcare and #postpartumexercises. The final images selected for the image exposure in this study were agreed upon by the study investigators. Selected images were eye-catching and chosen to represent a variety of accurate health information available in this public domain, including information about postpartum diet, physical activity, self-care and mental health.

### 2.2 Questionnaire

**Figure 1:**
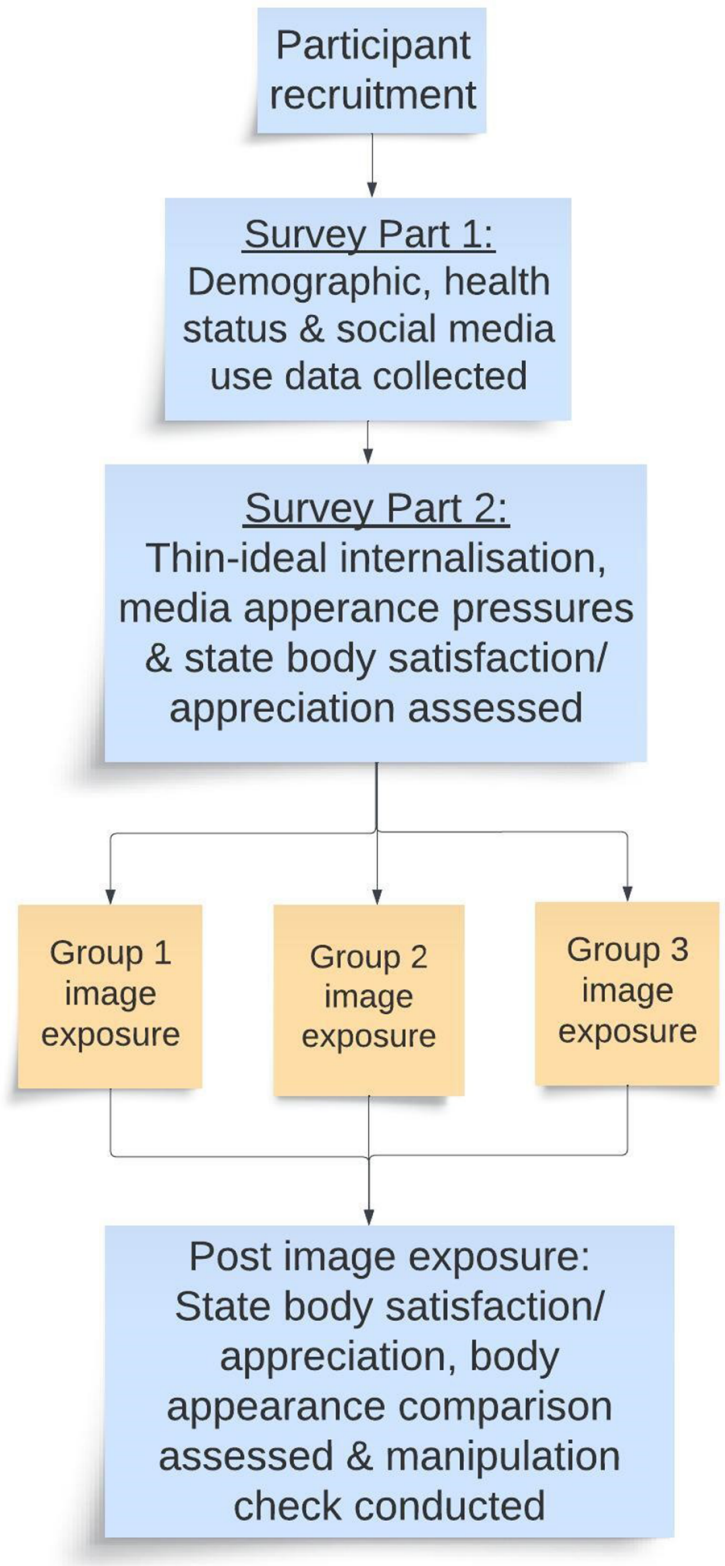
Study flow diagram.

All participants completed an identical questionnaire online (Supplement File 1) using REDCap electronic data capture tools hosted at The University of Sydney (16), which firstly collected demographic information (e.g., age, ethnicity, education), health status details (e.g., self-reported weight, height, known health conditions, any pregnancy complications) and social networking site use (Figure 1). Of note, one pregnancy complication listed as an option for women to select was “excessive gestational weight gain” which may have influenced how women feel about their bodies during completion of the survey.

The second part of the questionnaire firstly assessed thin-ideal internalization, media appearance pressures and state body satisfaction and appreciation. Participants were then directed via a link in the REDCap survey to the image exposure which was a YouTube clip. State body satisfaction and appreciation and body appearance comparison were then assessed after image exposure. Survey data was checked for repeat completion by examining date of birth and other demographic data by author MH. If data appeared to be repeated, one record was removed from the dataset.

### 2.3 Outcome measures

The primary outcomes for this study were change in state body satisfaction and state body appreciation from pre- to post- image exposure.

#### 2.3.1 State body dissatisfaction

State body dissatisfaction was measured using visual analogue scales (VAS) which are both suitable for repeated measurement and have demonstrated reliability and validity as a measure of body dissatisfaction (17). Participants were instructed to drag the marker along a line from 0 (none) to 100 (very much) to indicate how they feel “right now” for each item (weight dissatisfaction, appearance dissatisfaction). Scores on the weight and appearance dissatisfaction items were averaged to produce an overall body dissatisfaction score ranging from 0 to 100.

#### 2.3.2 State body appreciation

State body appreciation was similarly measured using VAS developed by Slater, Varsani, and Diedrichs (2017) (7). Participants were asked about their current feelings for three items, adapted as state versions of items contained in the Body Appreciation Scale (18). Scores on the three items were averaged to produce an index of state body appreciation ranging from 0 to 100.

#### 2.3.3 Thin-ideal internalization

Thin-ideal internalization was assessed prior to image exposure using the Thin/Low Body Fat subscale of the Socio-cultural Attitudes Towards Appearance Questionnaire (SATAQ-4) (19). Scores for five statements were averaged to produce an overall score ranging from 1 to 5, with higher scores indicating greater internalization of thin ideals. Internal reliability for this scale has previously been shown to be acceptable (5, 19).

#### 2.3.4 Media appearance pressures

Media appearance pressures (i.e., pressure applied by the media to achieve the societal ideal) were also assessed using the media pressures subscale of the SATAQ-4 (19). Scores for four statements were averaged to produce an overall score ranging from 1 to 5, with higher scores indicating greater appearance pressure from the media.

#### 2.3.5 Body appearance comparison

The degree to which participants engaged in appearance comparison while viewing the images was assessed (following image exposure) using the State Appearance Comparison Scale (20). The three items of the Scale were averaged to produce a measure ranging from 1 to 7, with higher scores indicating greater appearance comparison processing. Previous internal reliability has been shown to be good (5, 20).

### 2.4 Manipulation check

Group assignment was checked by asking participants to recall the proportion of images that were of women, and the proportion containing health information (none; one quarter; half; three quarters; all). These questions served as a manipulation check to ascertain whether participants who viewed images containing health information had noticed them.

### 2.5 Sample size

We aimed to recruit 300 participants (100 per group) based on a similar study in young women (5), where participants viewed Instagram images of thin or average-sized women containing either body positive captions or no captions. Recruitment of 100 participants to each group had 80% power to detect a difference in change in state body dissatisfaction score of 8 (based on mean = 50; standard deviation = 25; 5% Type I error rate). This clinically meaningful difference has been detected in previous studies assessing change in state body dissatisfaction following various media exposures (5, 17).

### 2.6 Statistical analysis

Statistical analysis was performed in SPSS v28.0 (SPSS Statistics for Windows, Armonk, NY). Descriptive analysis was performed. Chi-squared tests were conducted to assess differences between groups for demographic categorical data, e.g., age, ethnicity, health status and pregnancy complications and one-way ANOVA was used to assess differences between groups for continuous data before and after image exposure, as well as the changes in state body satisfaction and appreciation from pre- to post-image exposure.

Sensitivity analyses were also conducted to examine the impact of pregnancy, attention to the image exposure (i.e., correctly responding to manipulation check questions) and postpartum duration (i.e., 0–12 months vs. 13–24 months) on thin-ideal internalization, media appearance pressures and state body comparison as well as state body satisfaction before and after image exposure and change from pre- to post-image exposure.

## 3 Results

In total, 308 participants completed the image exposure and questionnaire including pre- and post- assessment of state body satisfaction and appreciation. Characteristics of study participants are displayed in Table 2. The majority were aged 26–35 years and had a university and/or postgraduate degree, and approximately three quarters identified as being Caucasian. Almost all were in a relationship, and approximately half had one child. Body mass index was in the overweight category for all groups. Most women experienced no self-reported health risk or pregnancy complication. The most common self-reported pregnancy complications were gestational diabetes (19%) and excessive gestational weight gain (14%). The only significant difference between groups was recruitment method, with more women in Group 1 being recruited from social media platforms, and more women from Groups 2 and 3 being recruited from the Playgroup New South Wales text message and Newsletter. In total, 95% of participants reported having a Facebook, 93% Instagram, 39% LinkedIn, 22% TikTok 18% Twitter account. This also did not differ between groups.

**Table 2:** Characteristics of study participants.

	Group 1 *n* = 101	Group 2 *n* = 102	Group 3 *n* = 105	*P* value
Age group, *n* (%)				
18–25	1 (1)	5 (5)	5 (5)	0.375
26–35	65 (64)	62 (61)	71 (68)	
36–45	35 (35)	35 (34)	28 (27)	
46–55	0 (0)	0 (0)	1 (1)	
Ethnicity, *n* (%)				
Asian	17 (17)	7 (7)	13 (12)	0.245
Caucasian	72 (71)	77 (76)	78 (74)	
European	8 (8)	10 (10)	6 (6)	
Other	4 (4)	8 (8)	8 (8)	
Education, *n* (%)				
Secondary school	5 (6)	7 (7)	10 (10)	0.827
Trade certificate/diploma	18 (18)	22 (22)	19 (18)	
University degree	45 (45)	38 (37)	40 (38)	
Postgraduate degree	33 (33)	35 (34)	36 (34)	
In a relationship, *n* (%)	100 (99)	98 (96)	102 (97)	0.532
Recruitment from, *n* (%)				0.009
Friend	8 (8)	7 (7)	2 (2)	
Text message from PNSW	3 (3)	15 (15)	13 (12)	
PNSW Newsletter	28 (28)	41 (40)	38 (36)	
PNSW social media post	0 (0)	2 (2)	3 (3)	
Other social media	61 (60)	36 (35)	48 (46)	
Other	1 (1)	1 (1)	1 (1)	
Number of children, *n* (%)^a^				0.272
1	58 (57)	47 (47)	47 (45)	
2	29 (29)	40 (40)	43 (41)	
3	13 (13)	10 (10)	10 (10)	
4	1 (1)	4 (4)	5 (5)	
Age of youngest child, *n* (%)				0.626
<6 months	18 (18)	27 (27)	25 (24)	
6–12 months	29 (29)	29 (28)	31 (30)	
13–24 months	54 (54)	46 (45)	49 (47)	
Pregnant, *n* (%)	8 (8)	4 (4)	2 (2)	0.109
BMI, mean ± SD	26.2 ± 6.6	26.1 ± 6.0	26.8 ± 6.2	0.709
Health risk present, *n* (%)				
Smoking	3 (3)	4 (4)	1 (1)	0.390
Obesity	19 (19)	16 (16)	23 (22)	0.520
High alcohol consumption	2 (2)	4 (4)	2 (2)	0.589
High cholesterol	1 (1)	1 (1)	5 (5)	0.108
High blood pressure	4 (4)	3 (3)	3 (3)	0.885
Diabetes	2 (2)	0 (0)	0 (0)	0.127
None	77 (76)	76 (75)	72 (69)	0.427
Other	1 (1)	2 (2)	3 (3)	0.627
Pregnancy conditions, *n* (%)				
HDP	11 (11)	7 (7)	14 (13)	0.306
GDM	21 (21)	20 (20)	18 (17)	0.793
T1D or T2D	1 (1)	0 (0)	0 (0)	0.358
EGWG	16 (16)	15 (15)	13 (12)	0.769
None	56 (55)	60 (59)	62 (59)	0.844
Other^b^	4 (4)	7 (7)	3 (3)	0.362

ATSI, Aboriginal and/or Torres Strait Islander; BMI, body mass index; EGWG, excessive gestational weight gain; GDM, gestational diabetes mellitus; HDP, hypertensive disorder of pregnancy; n, number; PNSW, Playgroup NSW; SD, standard deviation; T1D, type 1 diabetes; T2D, type 2 diabetes.

^a^ 1 missing in Group 2.

^b^ Most “Other” pregnancy complications were the condition Hyperemesis Gravida.

The majority of participants from Group 1 (no health information) correctly reported that all images were of women (92%), and that no images contained health information (81%). In Group 2, most participants correctly identified that about one quarter of the images contained health information (75%) while 47% and 42% reported that all or three quarters of the images were of women, respectively. In Group 3, 67% correctly reported that half of the images contained health information, whereas only 44% correctly reported that half the images were of women (31% reported that three quarters were of women, and 19% reported that all were of women).

### 3.1 State body satisfaction and state body appreciation

State body satisfaction and appreciation was not different between groups, either before or after image exposure (Table 3). Similarly, neither state body satisfaction, nor state body appreciation changed from pre- to post-image exposure in any study group and the change did not differ between groups. Thin-ideal internalization, media appearance pressures and state body comparisons also did not differ between groups (Table 3). Internal reliability was acceptable for thin-ideal internalization (*α* = .79), excellent for media appearance pressures (*α* = .94) and excellent for body appearance comparison (*α* = .94).

**Table 3:** Body satisfaction and body appreciation before and after image exposure.

	Group 1 *n* = 101	Group 2 *n* = 102	Group 3 *n* = 105	Degrees of freedom	F	*P* value
Prior to image exposure						
Thin-Ideal Internalization*	2.9 ± 0.9	3.0 ± 0.8	2.9 ± 0.9	2, 304	0.346	0.599
Media Appearance Pressures	3.4 ± 1.1	3.3 ± 1.3	3.3 ± 1.1	2, 305	0.456	0.968
State body satisfaction	42.7 ± 26.5	45.2 ± 26.5	42.8 ± 23.8	2, 305	1.213	0.732
State body appreciation	58.4 ± 24.7	59.7 ± 23.3	62.9 ± 24.1	2, 305	0.659	0.385
Following image exposure						
State body satisfaction	45.2 ± 27.6	46.5 ± 27.6	43.3 ± 25.4	2, 305	1.172	0.697
State body appreciation	59.5 ± 24.5	60.7 ± 25.5	63.3 ± 23.7	2, 305	0.837	0.525
State body comparison	3.8 ± 1.7	3.8 ± 1.7	3.8 ± 1.7	2, 305	0.921	0.986
Change from pre to post						
State body satisfaction	2.4 ± 15.1	1.2 ± 12.8	0.5 ± 13.5	2, 305	0.517	0.597
State body appreciation	1.0 ± 11.6	1.0 ± 10.6	0.3 ± 11.4	2, 305	0.127	0.881

* Data missing from 1 participant.

In sensitivity analyses our findings were the same whereby thin-ideal internalization, media appearance pressures, state body comparison and state body satisfaction and appreciation did not differ between groups when we removed the data from the 14 pregnant participants, nor when we excluded women who were more than 12 months postpartum, nor when we removed those who “failed” the manipulation check (data not shown).

## 4 Discussion

Our findings suggest that exposure to body-focused imagery, typical of what is commonly seen on Instagram targeting postpartum women (15), did not reduce state body satisfaction or appreciation. Furthermore, including postpartum health-information-focused images, in conjunction with body-focused images, did not influence state body satisfaction or appreciation compared with body-focused images alone.

We did not see expected reductions in state body satisfaction and appreciation following exposure to body-focused imagery. These findings contrast with the body of evidence demonstrating that Instagram use by young women, and viewing “idealized” imagery, is associated with worsening body satisfaction (4–7). However, some previous studies (4, 7) were conducted cross sectionally, and were not assessing differences in state levels of body satisfaction and appreciation. It is possible that changes in state body satisfaction and appreciation may have been detected in the present study if exposure to the images was longer, or if women were exposed to such imagery on multiple occasions.

Previous published studies showing negative impacts of body-focused imagery exposure were in young women (4–7), and not in postpartum women specifically, suggesting a potentially protective effect of the pregnancy experience and postpartum state on the effects of body-focused imagery on body satisfaction. Although we do not know whether images used in our experimental study were altered using filters or similar, it is possible that, due to their high social networking site use (2), postpartum women have become “desensitized” to the effects of photoshopped imagery on body image. Additionally, it is possible that their typical social networking site use has included exposure to, and increased awareness of, the body pride movement which may have empowered women to be less affected by exposure to this body-focused imagery (21). Regardless, our findings were unexpected, and would need confirming in future studies.

Women in our study were up to 2 years postpartum. This was to align with previous research which suggests that body dissatisfaction increases with time since child birth (10). Alternatively, other research suggests that body dissatisfaction may be heightened within 12 months of giving birth, when women may have expected to have lost weight gained during pregnancy, and hence may be more vulnerable to idealized imagery (13). This is in comparison to the potentially reduced vulnerability in late pregnancy, when women may be experiencing a new awareness of the functionality of their bodies (22). Nevertheless, our sensitivity analysis indicated that postpartum duration (0–12 months vs. 13–24 months) did not impact our findings.

Further research is required to investigate how delivering health information on social networking sites may impact women's physical and psychological health. A 2023 scoping review identified only three previous studies that have assessed the effectiveness of health information delivered on social networking sites to postpartum women,^1^ highlighting the need for more research. Our preliminary findings are reassuring, suggesting that encouraging postpartum women to access health information via social networking sites may not negatively influence their body satisfaction and appreciation. However, there are several challenges in conducting an intervention via social networking sites, including encouraging women to increase their screen time use, which may not be good for their overall health (23, 24). Future interventions should include collaboration with consumers (i.e., postpartum women) and the health promotion sector, to design effective health promotion strategies that are acceptable, modifiable and safe for delivery via social networking sites.

In the 2023 scoping review,^2^ none of the three studies assessing the effectiveness of health information delivered on social networking sites to postpartum women utilized Instagram for intervention delivery. However, 93% of participants in the present study report holding an Instagram account. Intervention designers need to consider that social media popularity and usage patterns are constantly evolving, and will need to monitor this and develop interventions that are able to be adapted to suit the most accessible sites.

There are several strengths of our research including recruitment of a sufficient sample size, participant blinding, and assessment of body satisfaction and body appreciation using validated tools appropriate for repeated measures. Limitations include the non-randomized, quasi-experimental nature of our study which, although groups were similar at baseline, limits the interpretation of our findings. We recognize that the images women see on Instagram vary greatly depending on the feed curation, therefore it is unlikely that the entirety of an individual's feed is aligned with #postpartumbody. We also recruited a highly educated, mostly Caucasian sample of women that had to hold at least one social media account, so may not be representative of the postpartum population. Our inclusion of pregnancy complication “excessive gestational weight gain” may have affected body image scores due to the shame inducing nature of this terminology. Future studies should use more appropriately worded terms and avoid stigmatizing language. Furthermore, image quality during the study may have been affected by internet connectivity or access to good quality technical devices, which we did not consider in our analysis. It is also possible that assessment of body satisfaction/appreciation over time (e.g., hours, day, few days, week), or exposure to more images, or multiple sets of image exposure over subsequent days, or exposure to images in a more “Instagram-like” set up may have led to different results, or indeed differences in state body satisfaction and appreciation between groups. It is also possible that this short-term exposure could impact initially on other aspects of mental health including mood and depression which we did not assess in our study. Health-information-focused images used in this study did not undergo content analysis or similar which should be conducted in future studies to better characterize these images.

Our preliminary findings suggest that short-term exposure to body-focused imagery typically seen on Instagram targeting postpartum women may not alter state body satisfaction or state body appreciation. A randomized trial with larger sample, longer follow-up, repeated image exposure and assessment of other aspects of mental health is needed to confirm a lack of effect. Nevertheless, further research investigating whether an intervention providing health information to postpartum women via social media platforms improves health outcomes may be warranted.

## Data Availability

The raw data supporting the conclusions of this article will be made available by the authors, without undue reservation.

## Footnotes

^1^Henderson MJ, Gow ML. Impact of health promotion interventions conducted via social media on women’s health outcomes: a systematic review (Under review).

^2^See footnote 1.
