# Supplementary material for: Impact on women's body satisfaction of exposure to postpartum imagery on social media
Source: Front Digit Health. 2025 Mar 12;7:1379337. doi: 10.3389/fdgth.2025.1379337 (PMC11937002; doi:10.3389/fdgth.2025.1379337)
Supplement: Supplementary file 1 [file Supplementaryfile1.pdf]

Supplementary File 1. Study Questionnaire

Study ID

____

ABOUT YOU

These first few questions are to find out about you, your background, and occupation. Questions marked with an asterisk (*) simply mean that they must be answered in order to continue.

What age group are you in?

- ◯
- ◯ 18-25
- ◯ 26-35
- ◯ 36-45
- ◯ 46-55
- ◯ 56+
- ◯ Prefer not to answer

What ethnic group do you identify with? Please select one answer.
(Please select one answer)

- ◯ Aboriginal or Torres Strait Islander
- ◯ Caucasian
- ◯ Asian
- ◯ European
- ◯ Polynesian or Maori
- ◯ Prefer not to answer
- ◯ Other (please specify)

____

What is your highest level of formal education?

- ◯ Did not attend school
- ◯ Primary school
- ◯ Secondary school
- ◯ Trade certificate/diploma
- ◯ University degree
- ◯ Postgraduate degree
- ◯ Prefer not to answer
- ◯ Other (please specify)

____

What is your usual occupation/profession?

____

Are you currently in a relationship?

◯ Yes
◯ No
◯ Prefer not to answer

Where did you hear about this survey?

◯ Playgroup newsletter
◯ Social media (e.g. Facebook, twitter, Instagram)
◯ Friend
◯ Other (please specify)

____

How many children have you given birth to (20 weeks gestation and over)?	◯ 0<br>◯ 1<br>◯ 2<br>◯ 3<br>◯ 4<br>◯ 5 or more
What is the age of your youngest child?	◯ Less than 6 months old<br>◯ 6-12 months<br>◯ 13-24 months<br>◯ Older than 2 years
Are you currently pregnant?	◯ Yes<br>◯ No

ABOUT YOUR HEALTH

Height (cm)<br>Please ensure you answer to the closest centimetre	______________________________
Weight (kilograms)<br>Please ensure you answer to the nearest kilogram	______________________________

From the list below, which currently apply to you? (select all that apply)

☐ Smoking
☐ High BMI (obesity)
☐ Alcohol consumption
☐ High cholesterol
☐ High blood pressure
☐ Diabetes
☐ None of the above
☐ Other

____

During any of your pregnancies, did you have any of the following conditions (select all that apply)?

☐ High blood pressure disorder e.g. preeclampsia, chronic hypertension, gestational hypertension
☐ Gestational diabetes
☐ Pre-existing diabetes mellitus (Type 1 or Type 2)
☐ Excess pregnancy weight gain (more than 16kg gain if of normal weight at beginning of pregnancy)
☐ None of the above
☐ Other, please state

____

How would you rate your diet over the past month?
In the assessment of your diet, please consider your intake of fruits, vegetables, wholegrain breads and cereals, dairy and lean protein.

◯ Excellent
◯ Good
◯ Fair
◯ Poor
◯ Prefer not to say

How would you rate your level of physical activity over the last month?
In the assessment of your activity levels, please consider that current recommendations are that adults undertake at least 1 hour of low-intensity, or 30 minutes of moderate-to-high-intensity activity per day

◯ Excellent
◯ Good
◯ Fair
◯ Poor
◯ Prefer not to say

SOCIAL MEDIA USE

Do you have any social networking/media accounts?

◯ Yes
◯ No

Which social networking/media accounts do you have? (select all that apply)

☐ Facebook
☐ Instagram
☐ Twitter
☐ LinkedIn
☐ TikTok
☐ Other
☐ I do not have any social networking/media accounts

What 'other' accounts do you have?

____

How often do you access your Facebook account?

◯ More than once per day
◯ Once per day
◯ A few times per week, but not daily
◯ Once per week
◯ Less than once per week
◯ Prefer not to say

How many Facebook friends do you have?

◯ 0-10
◯ 10-50
◯ 50-100
◯ 100-500
◯ 500-1000
◯ 1000-2000
◯ More than 2000
◯ Prefer not to say

How often do you access your Instagram account?

◯ More than once per day
◯ Once per day
◯ A few times per week, but not daily
◯ Once per week
◯ Less than once per week
◯ Prefer not to say

How many followers do you have on Instagram?

◯ 0-10
◯ 10-50
◯ 50-100
◯ 100-500
◯ 500-1000
◯ 1000-2000
◯ More than 2000
◯ Prefer not to say

How often do you access your twitter account?

◯ More than once per day
◯ Once per day
◯ A few times per week, but not daily
◯ Once per week
◯ Less than once per week
◯ Prefer not to say

How many followers do you have on twitter?

◯ 0-10
◯ 10-50
◯ 50-100
◯ 100-500
◯ 500-1000
◯ 1000-2000
◯ More than 2000
◯ Prefer not to say

How often do you access your LinkedIn account?

◯ More than once per day
◯ Once per day
◯ A few times per week, but not daily
◯ Once per week
◯ Less than once per week
◯ Prefer not to say

How many connections do you have on LinkedIn?

◯ 0-10
◯ 10-50
◯ 50-100
◯ 100-500
◯ 500-1000
◯ 1000-2000
◯ More than 2000
◯ Prefer not to say

How often do you access your TikTok account?

◯ More than once per day
◯ Once per day
◯ A few times per week, but not daily
◯ Once per week
◯ Less than once per week
◯ Prefer not to say

How many followers do you have on TikTok?

◯ 0-10
◯ 10-50
◯ 50-100
◯ 100-500
◯ 500-1000
◯ 1000-2000
◯ More than 2000
◯ Prefer not to say

Why do you use social networking sites? (select all that apply)

☐ To connect with friends
☐ To meet people
☐ To enhance my profile
☐ For work opportunities
☐ To access health information
☐ To follow celebrities
☐ Other
☐ Not applicable, I do not use any social networking sites

In your opinion, how important is the visual quality of images on social networking sites?

Not at all important
Not important
Slightly important
Quite important
Very important
Not applicable, I do not use any social networking sites

PART 1: HEALTH INFORMATION ON SOCIAL NETWORKING SITES

In your opinion, what health issues are most important for mothers who have one or more infants (children aged less than 2 years)?

☐ Breastfeeding advice
☐ Nutrition for the mother
☐ Physical activity for the mother
☐ Mental health information
☐ Tips on infant feeding
☐ None of above
☐ Other

____

Have you ever accessed health information on social media?

◯ Yes
◯ No

What health information have you accessed on social media?

☐ Breastfeeding advice
☐ Nutrition for me
☐ Physical activity for me
☐ Mental health information
☐ Tips on infant feeding
☐ Information about a specific condition
☐ Prefer not to say
☐ Other

What 'other' health information have you accessed on social networking/media sites?

____

Would you like to have access to health information on social networking sites in the future?

◯ Yes
◯ No

On which social networking sites do you think you would be most likely to access health information?

☐ Facebook
☐ Instagram
☐ Twitter
☐ LinkedIn
☐ TikTok
☐ Other (please specify)

____

How likely would you be to access the following types of information on social networking sites?

	Highly likely	Somewhat likely	Not sure	Somewhat unlikely	Highly unlikely
Breastfeeding information	○	○	○	○	○
Nutrition information for mother	○	○	○	○	○
Physical activity information for mother	○	○	○	○	○
Mental health information	○	○	○	○	○
Infant feeding tips	○	○	○	○	○
Information about a specific health condition	○	○	○	○	○

How would you like social networking sites to communicate health information to women with infants? (select all that apply)

☐ Via influencers
☐ Via health organisations (e.g. NSW health)
☐ Via health professionals
☐ Via researchers
☐ Via marketing of products
☐ Other
☐ Not sure
☐ Not applicable, I would not want to access health information on social media

What 'other' ways would you like to receive health information on social media?

____

Do you have any other advice for health professionals trying to convey health information via social networking sites?

BODY IMAGE PRE- AND POST- EXPOSURE TO IMAGES ON INSTAGRAM

In this part of the study, we are investigating how women in the postpartum period view their own bodies and how viewing images on Instagram impacts on this.
Completion of this part of the study should take you about 20 minutes and will involve answering questions about your views towards your body before and after viewing a number of images captured from Instagram.

As previously outlined, if at any time while completing this survey you become distressed, please exit your survey and contact Dr Megan Gow via phone (0421 078 958) or email (megan.gow@health.nsw.gov.au). Dr Gow will provide assistance and discuss further avenues of support with you, such as a review by a General Practitioner or Clinical Psychologist. For medical assistance you can consult your General Practitioner or contact services, such as:

- **Perinatal Anxiety & Depression Australia (PANDA) on 1300 726 306 or visit their website at https://panda.org.au/**
- **Beyond Blue on 1300 224 636 or visit their website at https://www.beyondblue.org.au.**

The following questions ask about how you feel about your body.

How do you feel at this very moment about your body?	Extremely dissatisfied … Extremely satisfied<br>*(Place a mark on the scale above)*
How do you feel at this very moment about your weight?	Extremely dissatisfied … Extremely satisfied<br>*(Place a mark on the scale above)*
Despite my flaws, I accept my body for what it is	Disagree … Neutral … Agree<br>*(Place a mark on the scale above)*
My feelings towards my body are positive for the most part	Disagree … Neutral … Agree<br>*(Place a mark on the scale above)*
My self-worth is independent of my body shape and weight	Disagree … Neutral … Agree<br>*(Place a mark on the scale above)*
I want my body to look very thin.	◯ 1 = Definitely Disagree<br>◯ 2 = Mostly Disagree<br>◯ 3 = Neither Agree Nor Disagree<br>◯ 4 = Mostly Agree<br>◯ 5 = Definitely Agree
I want my body to look like it has little fat.	◯ 1 = Definitely Disagree<br>◯ 2 = Mostly Disagree<br>◯ 3 = Neither Agree Nor Disagree<br>◯ 4 = Mostly Agree<br>◯ 5 = Definitely Agree
I think a lot about looking thin.	◯ 1 = Definitely Disagree<br>◯ 2 = Mostly Disagree<br>◯ 3 = Neither Agree Nor Disagree<br>◯ 4 = Mostly Agree<br>◯ 5 = Definitely Agree
I want my body to look very lean.	◯ 1 = Definitely Disagree<br>◯ 2 = Mostly Disagree<br>◯ 3 = Neither Agree Nor Disagree<br>◯ 4 = Mostly Agree<br>◯ 5 = Definitely Agree
I think a lot about having very little body fat.	◯ 1 = Definitely Disagree<br>◯ 2 = Mostly Disagree<br>◯ 3 = Neither Agree Nor Disagree<br>◯ 4 = Mostly Agree<br>◯ 5 = Definitely Agree
I feel pressure from the media to look in better shape.	◯ 1 = Definitely Disagree<br>◯ 2 = Mostly Disagree<br>◯ 3 = Neither Agree Nor Disagree<br>◯ 4 = Mostly Agree<br>◯ 5 = Definitely Agree

I feel pressure from the media to look thinner.	◯ 1 = Definitely Disagree<br>◯ 2 = Mostly Disagree<br>◯ 3 = Neither Agree Nor Disagree<br>◯ 4 = Mostly Agree<br>◯ 5 = Definitely Agree
I feel pressure from the media to improve my appearance.	◯ 1 = Definitely Disagree<br>◯ 2 = Mostly Disagree<br>◯ 3 = Neither Agree Nor Disagree<br>◯ 4 = Mostly Agree<br>◯ 5 = Definitely Agree
I feel pressure from the media to decrease my level of body fat.	◯ 1 = Definitely Disagree<br>◯ 2 = Mostly Disagree<br>◯ 3 = Neither Agree Nor Disagree<br>◯ 4 = Mostly Agree<br>◯ 5 = Definitely Agree

The next step in this survey is to click on the link below. The link will take you to a slide show where you will be exposed to a number of images that have been captured from Instagram. The slide show will go for xx minutes. Before clicking the link, please ensure that you have a good internet connection, and that you have xx minutes of uninterrupted time to view all images, if possible.

[Attachment: "Arm 1.mp4"]

How many of the images did you view uninterrupted?	◯ none<br>◯ about one quarter<br>◯ about half<br>◯ about three quarters<br>◯ all of them
How many of the images included some form of health information?	◯ none<br>◯ about one quarter<br>◯ about half<br>◯ about three quarters<br>◯ all of them
How many of the images were of women?	◯ none<br>◯ about one quarter<br>◯ about half<br>◯ about three quarters<br>◯ all of them
How thin, on average, were the images of the women you viewed?	not at all thin — very thin<br>*(Place a mark on the scale above)*
How attractive, on average, were the images of the women you viewed?	not at all attractive — very attractive<br>*(Place a mark on the scale above)*

To what extent did you think about your own appearance when viewing the previous images?	◯ no thought<br>◯ rarely thought<br>◯ occasionally thought<br>◯ sometimes thought<br>◯ frequently thought<br>◯ usually thought<br>◯ a lot of thought
To what extent did you compare your overall appearance to the appearance of the women in the previous images?	◯ not at all<br>◯ rarely<br>◯ occasionally<br>◯ sometimes<br>◯ frequently<br>◯ usually<br>◯ a lot
To what extent did you compare specific body parts to those of the women in the previous images?	◯ not at all<br>◯ rarely<br>◯ occasionally<br>◯ sometimes<br>◯ frequently<br>◯ usually<br>◯ a lot
How do you feel at this very moment about your body?	Extremely dissatisfied — Extremely satisfied<br>*(Place a mark on the scale above)*
How do you feel at this very moment about your weight?	Extremely dissatisfied — Extremely satisfied<br>*(Place a mark on the scale above)*
Despite my flaws, I accept my body for what it is	Disagree — Neutral — Agree<br>*(Place a mark on the scale above)*
My feelings towards my body are positive for the most part	Disagree — Neutral — Agree<br>*(Place a mark on the scale above)*
My self-worth in independent of my body shape and weight	Disagree — Neutral — Agree<br>*(Place a mark on the scale above)*
Comments	________________________________

Thank you for taking part in our study. If you know of any other mums who would also be interested in participating, please send them the flyer with the QR code. We really appreciate your assistance in helping us reach eligible participants.

If you would like to provide your email address to enter our prize draw and/or receive the results of this survey when they are available, please go to the following link [ENTER/ADD PART 4 LINK HERE]
